# Testing the public’s response to receiving severe flood warnings using simulated cell broadcast

**DOI:** 10.1007/s11069-022-05241-x

**Published:** 2022-02-16

**Authors:** Kate R. Smith, Silvia Grant, Robert E. Thomas

**Affiliations:** 1 Head of User Research, Strategic Command, Ministry of Defence, Whitehall, London, SW1 2HB UK; 2 Energy and Environment Institute, University of Hull, Cottingham Road, Hull, HU6 7RX UK

**Keywords:** Common alerting protocol, Cell broadcast, Emergency, Behavioural insights, Mobile alerting, Flood, Messaging, Warnings

## Abstract

European Governments must implement a public alerting system to reach mobile phone users affected by major emergencies and disasters by June 2022. Cell Broadcast is used to issue emergency alerts in several countries but has not yet been introduced in the UK. This paper presents the results of a joint research exercise that explored recipients’ responses to cell broadcast messages that warned of floods of varying certainty, severity, and urgency. We adopted a mixed-methods approach employing semi-structured questions and focus groups to assess the perceptions of 80 workshop participants who received simulated emergency alerts on pre-prepared handsets. Our results suggest that although emergency alerting is welcomed, it is necessary to provide accurate and verifiable information, address accessibility challenges, and state location clearly and understandably. This life-saving technology, if used aptly by not over-alerting, specifying the specific urgency, certainty, severity and location of the flood risk, has the real potential of upgrading flood warnings in the UK.

## Introduction

Article 110(2) EECC (European Electronic Communications Code) mandates that ‘by June 2022, every EU member state must implement… a public warning system that can reach mobile phone users affected by major emergencies and disasters’ (EU 2018). The UK Cabinet Office’s Civil Contingencies Secretariat has trialed different approaches to mobile alerting that would target members of the public in an area impacted by an emergency (Cabinet Office 2014). The need for such a service was reinforced by large-scale flooding in England in February 2020, and in the near-concurrent move towards a national coronavirus lockdown; this latter crisis resulted in government contacting citizens directly on their mobile phones (Grant 2020). The Cabinet Office established that there is a case for such a national mobile warning alert scheme (DCMS 2019) after conducting previous trials in 2013–14.^1^

The Environment Agency (EA) provides the strategic overview of the management of all sources of flooding and coastal erosion and has operational responsibility for managing the risk of flooding from main rivers, reservoirs, estuaries, and the sea in England. Their opt-in, SMS-based, Flood Warning Service has been in operation since 1996, giving them considerable experience in creating and sending emergency messages to the public. Flood Alerts are issued to warn people of the possibility of flooding and encourage them to be alert, stay vigilant and make early/low impact preparations for flooding. They provide advance notice of the possibility of flooding and may also be issued when there is less confidence that flooding will occur. Flood Warnings are issued when flooding is expected to occur and to alert people that they should take action to protect themselves and their property. In England, Environment Agency data shows that 15.6% of residential properties are subscribed to receive a notification if/when a Flood Warning is issued within their designated Flood Alert or Flood Warning Area (EA 2021). During the widespread flooding across England in February 2020, the Flood Warning Service issued over 1.4 million telephone warnings, 500,000 texts, and 560,000 emails. In the same event, the EA’s assisted digital channel, Floodline, took over 20,000 calls to their recorded message service and handled over 6000 live calls. However, neither the Flood Warning Service nor Floodline can reach all of those at highest risk during flood events (GOV.UK 2020), such as those travelling and visitors who may not be aware of a flood hazard.

Cell Broadcast has already been identified as a good candidate to match the emergency alerting needs of the EA (Cabinet Office 2014). In contrast to Short Message Service (SMS) messages, CB messages are broadcast directly and simultaneously from specified cells on mobile masts to multiple mobile telephone users. They can be sent to a range of cells/masts covering a wide area, or they can be targeted to specific areas covered by individual cells. CB messages can be sent as a single transmission or they can broadcast over an extended period of time. They do not require opt-in and no information is sent back to the sender. CB is not subject to network congestion. Any mobile phone in the area of the target mast, or travelling through its coverage area, will receive a CB message, potentially reaching millions of handsets near instantaneously. CB messages appear on mobile handsets in a variety of manufacturer-specific formats: handsets may vibrate and make a specific, very loud alert tone that is reserved only for this type of broadcast. Some will also read the message aloud.

**Fig. 1:**
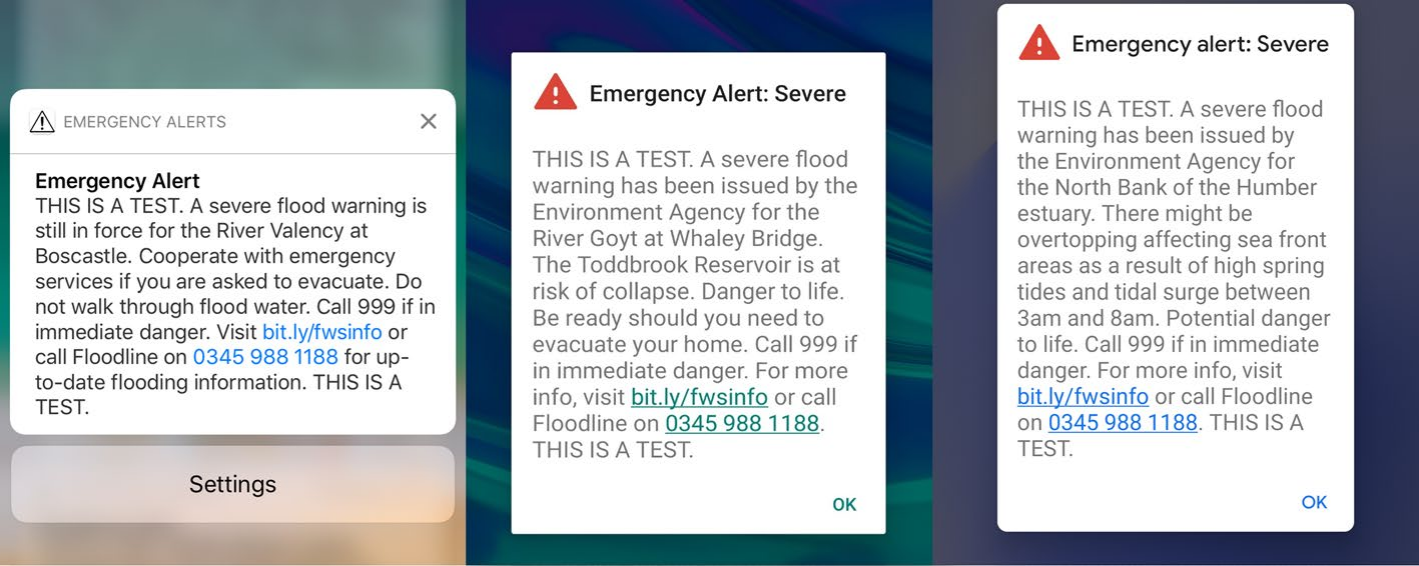
Screenshots of a cell broadcast messages used in research. *Source*: Fujitsu (2019)

Because of its expertise in the field of emergency alerting, the EA has been working with the Cabinet Office, mobile network operators, Fujitsu, and the University of Hull to test both the technology and users’ behavioural insights. Because they present a novel and challenging recipient experience for the naïve user, our research seeks to understand more about the public’s reactions to, ideas about, and actions leading from, emergency flood warnings sent by CB. The present paper reviews existing work on emergency alerting and CB and then outlines the theoretical approaches that underpin our research design. It then presents results of a series of on-campus workshops in November 2019 that used pre-recorded cell broadcast messages to simulate a live cell broadcast (e.g. Fig. 1) on handsets provided by Fujitsu. These results are discussed, along with directions for future research.

### Emergency messaging and cell broadcasting: research context

The literature around CB and emergency alerting is broadly formed of two types. The first consists of official reports and papers published by governments, agencies and researchers in the course of adopting CB. The second concerns behavioural reactions to emergency-specific alerts delivered in a range of media and for a range of hazards.

The present study is informed by the official government output of the UK’s early technology trials, with a focus on end-user perceptions. The UK government trials of 2013–14 favoured SMS as the preferred technology for emergency messaging. Reviewing the Comparative Technology Analysis of the resulting report suggests, however, that this conclusion was weighted towards the expectations of Mobile Network Operators (Cabinet Office 2014), and that SMS’s hackability and spoofability were underestimated, jeopardising the validity of SMS-based systems (NASEM 2018: 71), whilst their reliable reach was overestimated (Brewster 2020).

Following improvements in handset technology and 4G reach, most government research on CB was conducted between 2005 and 2011, when it became a viable option for public warning systems in conjunction with the integration of Common Alerting Protocol thresholds with XML from the mid-2000s (OASIS 2005). Notably, New Zealand published a report providing best practise for writing short warning messages for the public to achieve a desired behavioural response (Potter 2018). Australia shared best practise around ‘choosing your words’ (Australian government 2018), particularly with relevance to natural disasters, including advice on how to maximise message impact for non-English speakers. In the wake of the Victoria bush-fires disaster of 2009, the Council of Australian Governments agreed to urgently explore Emergency Alerts. This research considered whether first responders and government agencies felt that issuing of alerts happened efficiently and asked whether end-recipients of messages were satisfied that they had been provided with sufficient information (Torrens Resilience Institute 2011). Whilst Emergency Alert users were satisfied overall with the Australian messaging system, its effectiveness was contingent on several variables including community preparedness, quality of information, and timeliness of messages in a complex multimedia environment. The research noted that ongoing community education was necessary, particularly in regions prone to bushfires, floods and other geohazards.

In addition to this, significant contributions to knowledge about emergency alerting have been associated with the development of wireless emergency alerts (WEA), the USA’s name for CB. Following the development of the Integrated Public Alert and Warning System (IPAWS) in the mid-2000s, research has continued to refine and understand the impact of citizen alerting for a range of hazards in the USA. Early research (e.g. Mileti and Sorensen 1990; Sorensen 2000) noted that alerts should contain five key elements in order to motivate appropriate and timely response: hazard, location, guidance, time, and source. Lindell and Perry (2012)  proposed the Protective Action Decision Model (PADM) as a theoretical framework to assist understanding how people respond to environmental hazards and disasters. It attempts to capture the timeline from the receipt of external information (e.g. an alert or warning) to enacting a behavioural response via three pre-decisional processes (exposure, attention, comprehension), three core perceptions (threat, protective action, stakeholder) and three behavioural responses (information searching, protective response, and emotion-focussed coping) (Lindell and Perry 2012).

Bean et al. (2015) reviewed literature in the fields of public warning research, crisis communication and health communication, identifying research approaches to improve Wireless Emergency Alerts. Despite this growing body of work, strategies to optimise location communication and behavioural action are undetermined, as are the ideal repetition frequency and length of public warning messages (Bean et al. 2015). All of these challenges—and those of authoritativeness, personalisation and accessibility—need to be met within a fixed character limit (Bean et al. 2015). A comprehensive review of emergency alerting systems undertaken in the late 2010s summarised research on cell broadcast focussing particularly on WEA research conducted by academics supported by the Department of Homeland Security (DHS) (NASEM 2018).

This summary highlights that the majority of research has focused on experimental learning about participants’ reactions to messages. Research gaps identified in the NASEM review include proposals to develop more sophisticated integration of emergency messaging with other technologies, specifically with household WiFi-enabled devices, in an ‘integrated alert and warning ecosystem’ (NASEM 2018: 48). This proposal itself depends upon the in-filling of knowledge gaps about key issues addressed in the present research: how should information be linked, what kind of information be linked to, how do people understand location information, how can messages be made accessible, and what is the role of disaster alerting education, and to what extent should messages be personalised. More detailed research about user perceptions of (purposively intrusive) emergency alerts by Yoder-Bontrager et al. (2017) suggests that the susceptibility of messages to individual user preference necessitates personalisation. Their focus group research framed with PADM (Lindell and Perry 2012) indicated that recipients want to receive information about space and/or time in different ways, with divergence in the range of likelihoods, severity and urgency at which warnings would be welcomed. With a particular emphasis on learning about user perceptions, our research foregrounds user needs in terms of understanding these key issues.

More recently, Doermann et al. (2021) also used the PADM to identify best practise for crafting short messages about imminent threats. This best practise was then incorporated within a message creation tool by prompting the message creator to answer 15 questions. Doermann et al. returned to the familiar problem of milling that is a consistent theme in emergency messaging research (see for example Wood et al. 2018): if the recipient is uncertain about any of the content received or about how they should proceed, they will seek additional information which may delay taking protective action.

## Approach, methodology and participants

### Approach

Echoing Jagtmann (2010), user perception forms a central part of the theoretical underpinnings of the present study. The technology’s response (such as how much time is involved for the handsets to receive the broadcast from the mast, which model reacted first, and which handset vibrated the loudest) can be analysed experimentally. User and recipient perceptions, however, are dependent on individually constituted systems of intersubjective beliefs and values. The implication of this is that participants’ reactions to the technology’s materiality will impact the success of different modes of communication used for sending emergency alerts.

In terms of establishing a theoretical context for our approach, we follow Jackson and Sorensen’s (2006) elucidation of the state’s role as constructivist actor. As products of human invention, systems of nation-state and governance exist only as an intersubjective awareness amongst people. Constructivism holds that this awareness is a set of ideas, a body of thought, or a system of norms, that has been purposively arranged by particular people at a particular time and place (ibid 162). It follows that the citizen’s awareness of risk, particularly risk as defined by the Government, is not simply measurable on a linear scale: risk perception is modulated by systems of ideas, thoughts, and norms that are subject to contextual variation (Slovic 1992). As such, opinions about risk are informed by intersubjective beliefs; as beliefs change, systems of nation and governance (in this case of risk-based alerting) change too.

In parallel to this, perceptions about the ‘problem of risk’ (Lash and Wynne, in Beck 1992) are both diminished and amplified by physical and socio-cultural factors (Kasperson et al. 1988; Thistlethwaite et al. 2018). What we know about hazards and how we experience them impacts our sense of their riskiness (Viglione et al. 2014; Fielding and Hornsey 2016; Hamilton-Webb et al. 2019); how vulnerable we are to them is affected by our socio-economic agency, as is our capacity to respond to warnings about them (Botzen et al. 2009). The inverse association of wealth/agency/resource and the impacts of risk has been well described both theoretically and empirically (Phifer 1990; Beck 1992; Kasperson 1992; Zimmerman 1993; Fothergill 1996; Meredith et al. 2007; Tapsell and Tunstall 2008; Lujala et al. 2015; Stephenson et al. 2015; Walker-Springett et al. 2017); risk (and actions taken in response to it) is thus understood as socially constructed and mediated.

### Methodology

Our research binds together the theoretical and methodological frameworks mentioned above, focussing on the narrative constitution (Schmitt 2018) of participants’ responses. Based on a robust, iterative approach to qualitatively coding our research data, we correlate these meaning-making narratives with demographic data to determine whether there are patterns of response within our research cohort.

As this was a collaborative research exercise, our methodology was shaped by the policy-defining needs of our research partners; their focus in these exploratory trials was on end-user perceptions and behaviour. Bearing these priorities in mind, we devised a research exercise combining focus group sharing, responsive writing, and participatory polling. Within this approach, we adopted a linguistic constructivist theoretical framework in order to derive the most value from participant’s responses. This approach is predicated on discursive ontology’s understanding that the language with which ideas are expressed is constitutive of what is brought into people’s everyday reality (Hansen 2006). In practise, this allows us to explore the answers of respondents fully without constraining answers to set values, as seen in much of the literature around user perceptions and behaviour (e.g. NASEM 2018).

Our research was carried out in a series of workshops conducted on a single day. Each workshop followed the same pattern, with the same questions and tasks repeated by each workshop cohort. Questions put to participants and response modes are found in Table 1. Facilitation was provided by Environment Agency, Met Office, DEFRA and University of Hull staff, with audio recordings made of focus group sections. These were later transcribed and cross-checked with notes made by in-room facilitators.

**Table 1:**
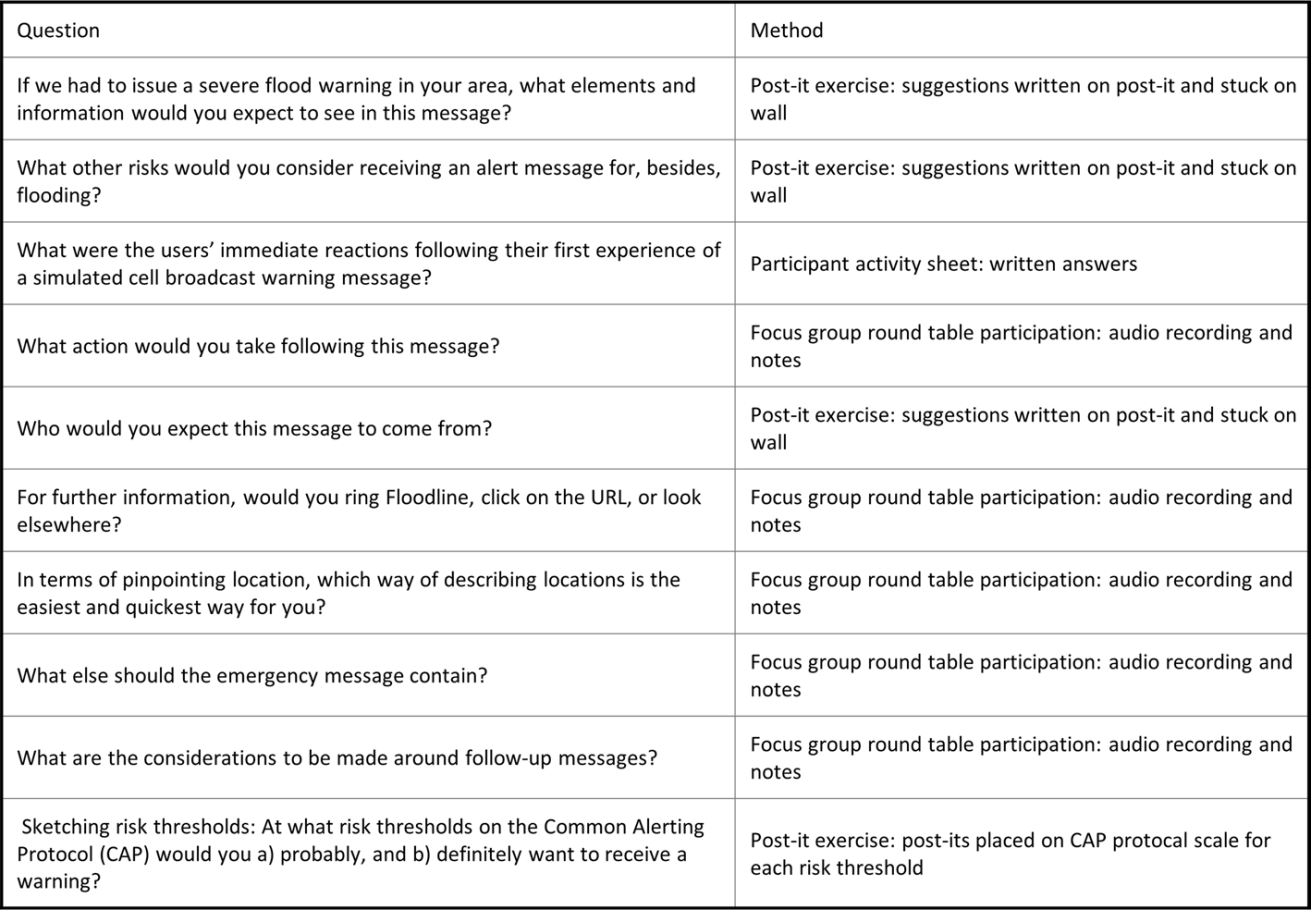
Question and answer modes

### Participants

We recruited 368 potential participants, from whom we selected a purposively stratified cohort of 80 that was representative of the University population (University of Hull 2019). We positively selected within this sample for individuals with experience of flooding. Whilst this means that the cohort differs somewhat from a nationally representative sample, it was a specific requirement of our research collaborators to curate a cohort that was representative of the University rather than the local population, which is somewhat less demographically diverse than many other high flood-risk areas of the UK (HCC 2021). It was also a priority for the Environment Agency to understand how people with experience of being flooded would react to a new kind of flood warning–part of the reason that Hull was chosen for this research is the relatively high proportion of people in the local community with personal experience of flooding within the past fifteen years (Ramsden 2021).

The mean age of the total sample cohort (hereafter referred to as participants) was 26. Just under two-thirds (60%) of the sample was female. The sample reflected the varying educational stages found on campus: 72.5% of participants were undergraduates, 15% were postgraduates, 5% were post-doctoral researchers or junior academics, and 7.5% were drawn from professional services. As the University of Hull and the EA are committed to improving equality, diversity, and inclusion we actively sought diverse representation in each workshop. Participants from black, Asian or minority ethnic (BAME)^2^ backgrounds made up 21% of the total sample cohort. We were unable to recruit a proportionate cohort of participants with disabilities: only 6% of our participants declared a disability during recruitment. Having positively selected for flood experience, the eventual percentage of all workshop participants who had experienced flooding was 18.75%. Implications of these decisions are discussed in Sect. 5 below.

Participants were allocated based on availability to one of three workshops run over a single day. Each workshop had the same tasks but featured different emergency message scenarios, based on real flood events (see Fig. 1). Using structured questions, each workshop (which was sub-divided into two focus groups) yielded activity sheets, photographic and physical artefacts from post-it note sessions, typed notes and audio recordings. Facilitators were deployed to each focus group to ensure consistency and to audio-record each session. Recordings were then transcribed and anonymized prior to further analysis. Questions and response modes can be seen in Table 1.

### Analytical strategy

Our analytical strategy focussed on identifying patterns and clusters in the frequency at which particular themes occurred across the dataset. Qualitative analysis was conducted using QSR NVivo; it consisted of iteratively coding keywords to identify themes, structured around the questions posed to participants. Conceptually-related sub-levels were aggregated from parent codes, allowing us to create a network of themes within the data, which in turn allowed us to identify both broad concept-level responses from the whole cohort and specific reactions at the level of the individual. The results of this coding were analysed using a series of matrix queries that compared case classifications (age, gender, flood experience, ethnicity, disability) against each theme. The results of these queries were then subjected to numerical analysis using MS Excel, including single factor ANOVA tests to establish significance where appropriate. Owing to the small sizes of each workshop group (< 35 in each group) most cross-tabulated results cannot be quantified as statistically significant using chi-square analysis. To support our numerical findings and to provide a qualitative visualisation of participant perceptions (Cidell 2010), we used QSR NVivo’s frequency analysis tools to generate word clouds relating to each theme. Where frequencies are reported in results these are counts of ‘text coded at’ rather than of participants.

## Results

### Message sending and content ideation tasks

At the start of each workshop, we invited participants to consider the range of emergencies for which an alert might be sent, and what elements such a message should contain. The most common response (34%, n = 66) indicated that being given direct instructions about what to do was important. This included being told where to go or avoid, who to contact for more help, and how to evacuate safely. Information about the location of the hazard was more of a priority than the severity or duration of the hazard. Whilst only 3% of participants (n = 5) noted the importance of making sure the message appeared to be genuine, our observational notes indicate that this was a recurrent theme in conversations during all three workshops; concern about the veracity of mass messaging recurs in later answers and reflects a general theme of moderate mistrust demonstrable in both immediate reactions and in anticipated milling behaviours. There was considerable support amongst participants for achieving authenticity, but little consensus about what would signify this.

Before the simulated messages were displayed, we asked participants to identify the hazards for which they might expect to receive an emergency message. Within a wide range of hazards, we noted some surprising themes: wildfires and earthquakes were the two most frequently identified geohazards, comprising 51% (n = 19) of geohazard-related suggestions. Flooding was only identified in 16% (n = 6) of responses despite Hull and the Humber region’s long-standing elevated flood exposure to a North Sea tidal surge; the National Risk Register identifies these kinds of floods as both the most severely impactful and the most likely of all UK geohazards (Cabinet Office 2017). Only 6% (n = 6) of participants referenced epidemics or pandemics as a hazard warranting an emergency alert.

### Focus group tasks

After these two introductory tasks, participants were invited to join either of the two focus groups in the room. A range of mobile handsets was distributed, each of which had a live screen capture of a cell broadcast emergency message pre-recorded in secure laboratory conditions. The messages were played simultaneously, to replicate as closely as possible the conditions of receiving a live message. Each handset given to participants responded to the message slightly differently, but the text was consistent within each workshop. The EA was particularly keen to vary the message content between each session. The three messages are shown in Fig. 1. Participants were asked to write down their immediate reactions to the message; they were not obliged to answer and the number of reactions they could record was not limited. As noted above, responses have, therefore, been quantified by coding frequency; some responses therefore include text coded at multiple themes.

Initial analysis of participants’ responses elicited three broad themes: reactions were cognitive, sensory or emotional. Table 2 summarises all reactions to the message receipt experience across all three workshops. Whilst our in-room observations emphasised the sensory impact of the simulated message such as participants wincing, moving their heads away from handsets or covering ears, data from participant activity sheets contained more cognitive reactions than sensory or emotional ones. Observations of the body language of participants indicated that the distinctive, loud and penetrating tone used for CB messages was jarring and unpleasant: participants physically recoiled from the handsets they were holding, blocked their ears and displayed facial expressions consistent with pain or discomfort (Prkachin 1992; Chen et al. 2018).

We postulate that the common sensory experience of participants has in many cases translated to a high number of what we have termed cognitive responses: visible signs of physical discomfort were nearly universal in the room, but the largest single category of reaction written down by participants is associated with comments about the message alert/tone/narration being attention-grabbing and/or effective. Although we intended this experiment to capture as unfiltered a ‘think out loud’ response as possible from participants, it is possible that the translation from sensory input to written output encouraged participants to abstract a cognitive judgement based on their sensory reaction.

**Fig. 2:**
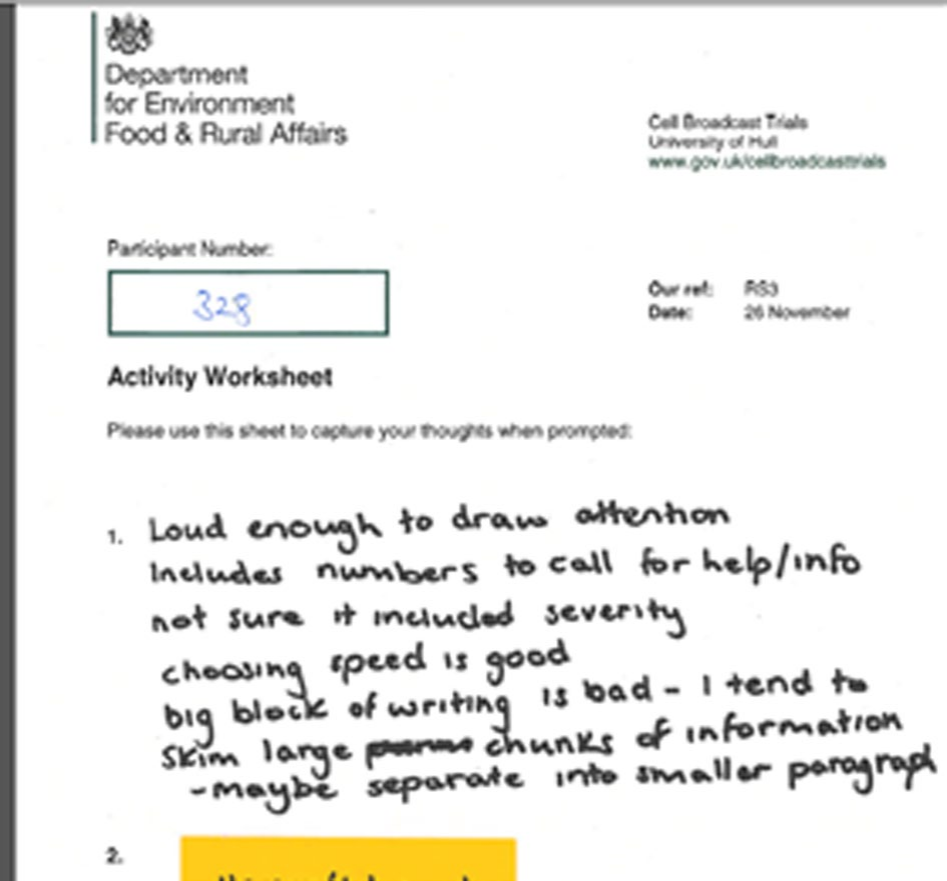
Extract of responses from participant 328

Within the sensory reactions that were written down, participants experienced negative reactions to both the volume and pitch of the alert tone and to the voice when messages were read aloud. Participants found that the voice read too quickly, was difficult to understand, and/or had an accent that was mistrusted or robotic. A negative reaction to the message volume or tone did not necessarily translate to a negative appraisal of the message as a whole. Figure 2 shows a participant’s comment from workshop 3, which is characteristic of responses as a whole.

The relatively small sample sizes limited the potential to calculate statistical significance for individual response themes and cohort variables. Using Single Factor ANOVA showed that age has a statistically significant impact on perception of loudness as negative (*F* = (1,6) 7.279, *p* = 0.04) and that flood experience, ethnicity and disability impacted people’s overall reporting of sensory (and wholly negative) reactions (*F* (1,10) = 8.273, *p* = 0.016).

As with sensory reactions, the emotional reactions recorded by participants were wholly negative. Participants reported feeling overwhelmed, panicked, scared and anxious; 37% felt overwhelmed by the message, while 60% were concerned about public or individual panic. Single-factor ANOVA analysis suggests a statistically significant relationship between feeling overwhelmed and gender, ethnicity and flood experience (*F*(1, 10) = 10.79, *p* = 0.008); disability status did not appear to impact the specific response of feeling overwhelmed but disabled participants were more likely to report individual panic. Similarly, although sample sizes are small, responses citing feeling overwhelmed were reported more than three times more frequently by female than by male participants, by people who had experienced flooding, and by BAME participants.

We characterised the third group of reactions as cognitive-based on the semiotic linking of themes as reflecting participants thinking about their reaction, rather than merely stating it. Thus, nearly half the responses characterised as cognitive note that the loudness and/or distinctiveness of the message makes it more effective. Extrapolation from their experience of the simulated message led some participants to consider the risks of sending such a message: some participants felt that such messages might cause public unrest, particularly if they were over- or misused. The issue of over-use was also linked by some participants to the issue of veracity or credibility, with some participants noting that over-use would render messages less convincing overall: ‘It should only be posted if it is an emergency…No other time because the more it is used, the less valuable it becomes…Only used in Red alerts [sic]’ (Participant 119).

Focus groups were next asked to verbalise the actions that they would take in response to an emergency message. From the eight themes identified in responses, four occurred more frequently than others: *prepare for evacuation*, *assess veracity/risk*, *contact people* and *click the link*, accounting for 92% (n = 137) of all reported actions. We noted that participants aged 18–21 were more likely than other groups to engage in milling by assessing the veracity of the message (although previous caveats about small sample size apply here as well), and that women were slightly more likely to prepare for evacuation than men (cf Enarson and Scanlon 1999 and Bateman and Edwards 2002), although differences were not significant. Participants with past experience of flooding were twice as likely to report preparing for evacuation than non-flooded participants (87% of all flooded participants vs 35% of all participants). Although each workshop was shown a different emergency scenario (see Fig. 1), we found no significant difference in anticipated actions between the groups (*F* (2,21) 0.937, *p* = 0.4).

We next asked participants what information they thought was missing from the message. Half the responses (n = 41) asked for more specific information of various kinds. Specificity was particularly important to people who had previously experienced flooding: 73% of requests for specific information compared came from to only 29% of requests for specific information came from people with flood experience although they represented 21% of the total cohorts. Younger participants were keen to see accessibility features included within the message: out of 24 suggestions to include accessibility, 20 were made by participants aged 18–21. These suggestions included multi-modal alerting using handset torch/flash, sound and vibration functions simultaneously, and relaying the message to linked accessibility aids such as screen readers.

The issue of post-message milling or information-seeking is characterised as an undesirable message receipt behaviour (cf Wood et al. 2018; Doermann et al. 2021), however, it is also widely accepted as inevitable (NASEM 2018:19). As the Environment Agency offers multiple flood warning channels it was important to understand which of these (or what else) participants would use. Our messages contained a shortened hyperlink to an EA website; the most common response (35%, n = 26) indicated that participants would click this link, but 8% (n = 6) indicated that they would not, citing concerns about security. Some participants would double-mill, verifying both the authenticity of the message and the detail of the hazard: ‘needs more authentic website link as could be confused w/ spam’ (participant 219); ’Google to see if more information online, call friends/family to see if they have heard about it’ (participant 319). The 18–25 age group were statistically more likely to use the link supplied than participants aged over 25 (χ^2^ = 10.16, pf = 1, *p* =  < 0.01). Sample sizes for other responses are too small to be reliable when determining whether age is an independent variable.

Willingness to call Floodline showed surprising patterns: of responses citing that they would definitely not call Floodline, 30% were made by previously flooded participants, despite them representing only 19% of all participants. As with other research involving Hull’s previously-flooded citizens (Ramsden 2021), the ongoing mistrust and disappointment after the flood events of 2007 and 2013 re-emerged as a topic for discussion during focus group sessions. In our discussions about Floodline, those who would use the service recalled positive experiences of the care and reassurance that Floodline operators had been able to provide in the past. Those who would not use it shared their experiences of lengthy wait times, and the frustration of seeking help from well-meaning but poorly informed call handlers who had little knowledge of the flooded area. The discussion around these experiences suggested that revisiting the experiences of people who sought help from Floodline and/or the Flood Warning Service would be beneficial to the EA.

The difficulty of identifying hazard location in locally meaningful ways poses a challenge for senders of short emergency messages. This difficulty is only enhanced by the divergence of individual meaning-making processes to do with place and space. The social construction of ‘location’ echoes that of ‘risk’: it is built through a combination of behavioural and psycho-social ideation. Unlike the construction of risk, however, the construction ‘place’ or location also has a physical element (Stedman 2003). Thus, physical landscape has a critical–if individually specific and nuanced–role in creating meaningful personal geographies. As the restricted length of emergency messages makes sharing information about the specific locations challenging (cf Bean et al. 2015) we asked how participants thought location information would best be communicated.

Within a broad range of responses, there were clear patterns in how participants wanted to be told about the specific location of a hazard: the most frequently identified theme indicated that participants would value links to maps (30% n = 31), and roughly equal segments suggested the need to provide either more specific details or indicate exposed postcodes (both 14% n = 15). Slightly fewer responses (12% n = 13) suggested relative distance from the hazard would be helpful. This result corroborates recent research that found the inclusion of maps helpful for increasing comprehension of short messages (Sutton and Kuligowski 2019). Participants who had been flooded were more likely to want information about potential evacuation routes or destinations as part of the message although small sample size makes statistical confidence tests unreliable. Like participants with disabilities, they were also more likely to suggest including maps. Female participants were more likely than male participants to suggest that relative distance should be included although again small sample sizes make χ^2^ an unreliable test of significance.

Participants were asked whether they would expect to or like to receive follow-up messages. There was enthusiasm for this possibility, with 61% (n = 22) of responses deeming follow-up messages to be necessary. Several responses indicated that messages should not be excessive, should include a ‘no longer in force’ message, and that follow-ups should be mentioned in the first message.

The scenarios shown to participants all originated from the Environment Agency, but the eventual adoption of cell broadcast as the UK’s emergency alerting technology will mean that messages come from a range of senders. We, therefore, asked participants to consider potential sources of emergency messages. Whilst the majority of responses named either specific or generic emergency services (39%, n = 49), the EA were cited in 24% (n = 31) of responses, followed by smaller proportions identifying central government and local government or city councils.

**Fig. 3:**
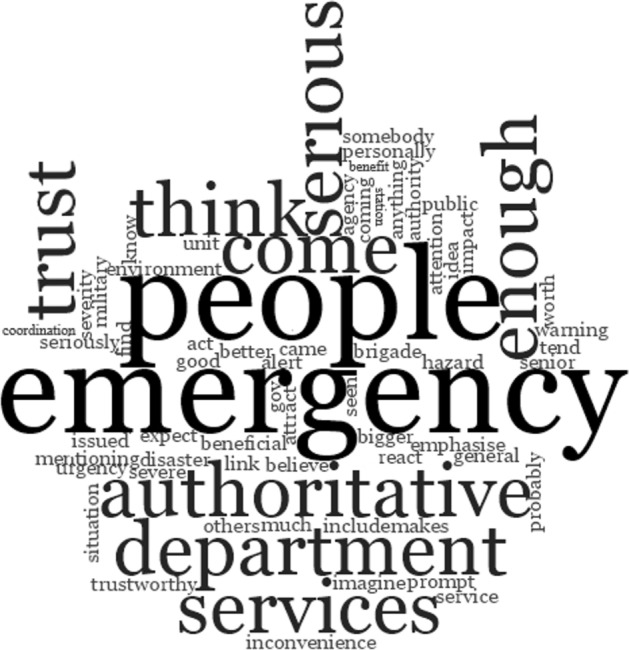
Word frequency cloud, top 500 words coded under 'Fire and rescue'

Responses from participants with flood experience or from BAME participants favoured messages coming from the Emergency Services; BAME participants were less likely to expect communications to come from the Environment Agency. Where specific services were mentioned, this was most frequently the Fire and Rescue service. Participants seemed to have a considerable degree of trust in this service: they were seen as having ‘more urgency… more like a disaster than an inconvenience’ (participant 101). A word-frequency query of the top 500 results for text coded under ‘Fire and Rescue’ reflects this positive association (Fig. 3). Comments during earlier discussions about the EA suggest that, although they are generally well recognised, participants had lower levels of trust in messages sent by them.

### Limitations of the research

We chose a cohort from self-selecting volunteers who responded to our call for participants, so participants may have been more motivated to think about flooding than would be case from random selection, although the exact content of the workshops was not revealed until participants were in the room.

Our final workshop cohorts reflect the demographics of the on-campus population at the University of Hull, which has a skew towards people aged 18–21, and which is more ethnically diverse than Hull and East Yorkshire (HCC 2021). To that end, however, we positively selected black, Asian and minority ethnic participants (BAME) to achieve a 20:80 balance between BAME and non-BAME participants that were more representative of the UK’s national demographic characteristics.

We were unable to recruit a nationally representative proportion of people with disabilities. Current estimates suggest that 19% of working-age adults in the UK have a disability (DWP 2021) but only 6% of volunteers indicated that they had a disability. Working to the EA’s equality, diversity and inclusion standards (DEFRA 2020), we, therefore, elected to positively select from this percentage of volunteers when populating our workshop cohorts, achieving a representation of 7.5% of the total cohort. Although this not ideal, the extent to which accessibility has become a mainstream consideration is reflected in the extent to which suggestions for making messages accessible came from non-disabled participants.

It was important to our research partners to foreground the experiences of people who had been flooded in the past. Though there is ample research supporting this choice (see for example Wind et al. 2013; Silver and Andrey 2014; Rickard et al. 2017; Thistlethwaite et al. 2018) which indicates that previous experience of environmental hazard has a complex relationship with the understanding of future hazards, we have been unable to confirm statistical significance across all themes and research questions because our focus group sizes were small.

Indeed, small cohort sizes in general limit the extent to which we have been able to infer statistical significance from our findings. This work was originally conducted as an exploratory exercise in conjunction with multiple non-academic partners. As an exercise in both knowledge exchange and academic research we prioritised our collaborators’ needs in our research design and our results are not therefore generalisable. The number of workshops we were able to run was constrained by the timescales required by collaborators. Furthermore, as all results were recorded as part of a group process, there is a possibility that results could have been skewed by group mobilisation, although facilitators endeavoured to minimise this possibility by using the same structure and wording during each session. Finally, recording and transcription difficulties led to gaps in the data record from one focus group in one workshop, such that 18 responses were excluded from our analysis.

## Discussion

By exploring the ways that people respond to and anticipate their actions in the light of emergency messaging, we have found a high level of support for robust, reliable, locally meaningful emergency messaging. There was a high level of support for CB as a mode of messaging because of its novel alert mechanisms (in contrast to Yoder-Bontrager et al. 2017). The profound attachment of many individuals to their mobile phone (Vincent 2006; Konok et al. 2016) coupled with participant comments such as ‘Losing control over my phone is inherently scary’ (participant 101) suggest that a fruitful avenue for further research could extend the experimental approach of Bean et al. (2016) by more directly considering the materiality of emergency warnings. As noted in NASEM (2018) the field of emergency warnings is inherently interdisciplinary, yet there is relatively little research on the subject that foregrounds methodologies more grounded in the social and communicative sciences. For example, the focus group discussions that revealed ongoing ambiguity to some flood risk management institutions could be expanded by investigating the pathways through which the particular cultural form of the ‘flood warning’ travels in specific directions (Briggs, 2021:73).

Our results reflect a considerable disjunction between the official UK government risk register and perceptions of risk by the public. The prominence given to risks that are relatively unlikely to occur in the UK (earthquakes and wildfires) supports broadly constructivist theories of risk understanding inasmuch as it seems likely that participants were influenced (in relation to wildfires at least), by the unfolding catastrophic fire season in Australia which was starting to be widely reported as our research took place. Given that only 6% (n = 6) of participants referenced epidemics or pandemics, there is a clear case for repeating this part of our research to test the correlation between media context and risk perception in the context of the coronavirus pandemic. If awareness of hazards correlates to exposure to hazard narratives, and thence to hazard preparedness, our results support previous arguments for public education as part of emergency planning across a wide range of emergency warning scholarship (Heath and Palenchar 2000; Gow 2009; Bean et al. 2014, 2015; Bean 2019). The public health crisis brought about by the emergence of COVID-19 in late 2019 only emphasises the strong relationship between public responsiveness, emergency planning and crisis resilience (Bean et al. 2021). If similar workshops were repeated with a post-COVID-19 cohort, we would anticipate a wide range of differences in risk sensitivity, behavioural expectations and perceptions of message authenticity.

In terms of meeting the needs of all emergency message recipients, our findings both confirm and extend those in previous research (Fothergill 1996; Enarson and Scanlon 1999; Bateman and Edwards 2002; Llewellyn et al. 2016) and point to the need for more disability-aware research, both generally in terms of demographic representativeness and specifically in addressing accessibility barriers, unequal impacts and lower resilience experienced by marginalised social groups. That disabled participants were significantly more likely to report individual panic on receipt of a CB message highlights the urgent need to better consider marginalised sections of the population in designing and operating civic structures. This includes recognising the particular difficulties faced by people with disabilities both when emergencies happen (Peek and Stough 2010) and in their aftermath.

Younger participants’ concern that more vulnerable people could be excluded from any emergency warning system is testament to the major social and legislative changes wrought by the long campaign for better recognition of the rights of people with disabilities. The field of disaster risk reduction for people with disabilities is a relatively new one (Llewellyn et al. 2016); good quality data on disasters and disability is relatively scarce (Villeneuve 2018). This points to the urgency of further work to bring people with disabilities into the emergency planning process- and also to ensure that implementation of emergency messaging technology is accessible to the whole community.

The differential impacts of additional vulnerability and sensitivity are a notable thread throughout our data. The cumulative impact of repeated flood experiences has already been well documented (see for example Wind et al. 2013; Lamond 2014; Stephenson et al. 2014; Foudi et al. 2017). During our workshops, participants with flood experience retold their own flood stories, noting how difficult it was to remain focussed on priority actions when under stress. Having clear, concise and specific information (with key content highlighted) took precedence for them, particularly as they were more likely to prepare for evacuation if instructed to. This finding provides end-user corroboration of previous studies that emphasise the need for clarity, concision and specificity when crafting emergency messages (Lachlan et al. 2007; Bean et al. 2014, 2015; Sutton et al. 2015; Kuligowski and Kimball 2018; NASEM 2018; Reynolds and Lutfy 2018; Sutton and Kuligowski 2019; Bean 2019).

**Table 2:** Reactions to cell broadcast message

Emotional reactions	Count	%	Sensory reactions	Count	%	Cognitive reactions	Count	%
Overwhelmed	13	28	Loud-negative reaction	27	54	Attention grabbing/effective	48	55
Public panic	11	24	Voice-negative reaction	13	26	Too much text	14	16
Individual panic	10	22	Generally unpleasant	10	20	Reading out is helpful	10	11
Scary	8	17	Total	50	100	Risk of negative response	9	10
Anxiety	4	9				Should not be used	4	5
Total	46	100				Risky for car drivers	3	3
						Total	88	100

Few drawbacks to CB messages were identified by participants, although three participants raised concerns about the potential risks to car drivers. Since these messages have the capacity to override handset settings, they could still be received by handsets in ‘drive’ mode. The impact of emergency messaging on drivers and other people in safety–critical situations needs to be better understood if effective mitigation measures are to be included in its deployment. This is clearly an area that merits further research.

Previous research has established that information seeking is a well-established response to official emergency warnings (Mileti and Sorensen, 1990; Sutton et al. 2019; Shklovski et al. 2008; Bean et al 2016). Our data suggest some avenues for future research about milling in different groups: on the one hand, participants felt that contacting a live helpline would give reassurance based on past experience but on the other, some recalled lengthy delays and a lack of additional local information. Future research could track Floodline user experience over time; the EA continues to improve its warning services, so historic perceptions of distrust may relate to outmoded technologies.

The benefits of including feedback mechanisms or reciprocity are emphasised in the recommendations made in NASEM (2018) but do not feature at all in our data. Furthermore, the UK government in part adopted cell broadcast over SMS because it is unidirectional. Apart from the problematic ethics of increasing recipient-to-sender surveillance, retro-fitting recipient monitoring or feedback would remove this benefit. The more general ethical challenges posed by the implementation of cell broadcast are also pertinent here. The implications of any move towards customisation, feedback, or integration with handset location technologies need to be carefully considered, as do the impacts of messages on more vulnerable recipients. As noted by Bean et al. (2015) the use of emergency alerting has outpaced research into its consequences and implications. It would seem prudent, therefore, for the ethics of extended emergency alerting to be properly explored before such integrations become commonplace.

The drive to exploit the geolocational capabilities of smartphones is understandable given the ongoing challenges posed by meaningfully identifying location in a message of limited length. Considerable discussion took place within each focus group around the merits or otherwise of using postcodes as a means of identifying the hazard location. On the one hand, some participants felt very strongly that postcodes would help them understand the relative proximity of the event, whereas others pointed out that they would not be familiar with postcodes other than their own. Other participants noted that, in the case of a catchment-wide scenario, a range of postcodes would be affected that could not easily be summarised. We add our voices to those calling for more research into improving hazard location communication (including Bean et al. 2015; Cao et al. 2017; Liu et al. 2017).

Participants’ thinking about follow-up messages both confirm and challenge previous findings. People with experience of flooding in the past were more sensitised and cautious around potential future floods (Mason et al. 2010; Stephenson et al. 2014, 2015; Foudi et al. 2017; Walker-Springett et al. 2017; Hamilton-Webb et al. 2019), but we found no evidence that marginalised communities are less likely to access or rely on official messaging (Lachlan et al. 2007; Meredith et al. 2007; Wray et al. 2008). Responses about potential message senders suggest a high level of trust in the emergency services across all demographic groups: as trust is a critical component in effective emergency messaging (Meredith et al. 2007), harnessing this high esteem could increase the chances of recipients engaging with emergency messages (Heath and Palenchar 2000; Meredith et al. 2007; Wei et al. 2018).

## Conclusion

CB is a technology that enables life-saving emergency alerting, which is of particular interest in severe flooding scenarios. Unlike traditional flood warnings, which are issued on a flood risk zone basis, it allows responsible agencies to dynamically warn people at risk in any location. Its distinctive reception and handset behaviour stimulates attention from end-users, as long messaging capability is not over-used, or under-delivered.

The mixed-methods approach that we employed highlighted important considerations such as concerns about community reaction to the emergency warning’s alert tone, progressive desensitisation of recipients to emergency messaging, the need to provide accurate and verifiable information, the requirement to state locations clearly, and explicit inclusion of vulnerable citizens. The constructivist notions underpinning these findings demonstrate that the reality of risk for participants is constituted by their personal interpretation of risk narratives. This study posits that this risk construction was affected by personal circumstances such as disability, gender, and flooding experience, as well as environmental factors, such as hazards covered in current affairs. This means that participants do not hold an objective view of risk levels. Rather, their risk appetite is constructed by complex factors which in turn shape the reality in which they live. This can be hard to quantify for bodies such as the EA when needing to issue clear warnings to the general public.

Further research is recommended to explore similar questions on different segments of the population such as end-users with protected characteristics and vulnerabilities, speakers of other languages. Given that smartphones near-universal accessories thought needs to be given to the messaging’s impact on vulnerable populations. As post-traumatic stress disorder is relatively common amongst people who have been severely flooded in the past (Asim et al. 2019), we need also to understand the impact that CB messages could have on people who have experienced major and/or repeated hazards. It is also recommended that the different settings in which users might receive a CB message are also investigated. This technology is meant to be disruptive; it is therefore also important to consider its impact in settings where disruption might have an adverse impact such as in hospitals and schools, and on drivers.

This life-saving technology if used appropriately by not over-or under-alerting, identifying the specific details and location of hazards, has the potential to improve flood warnings in the UK, particularly in areas which are not currently covered by the EA flood messages.

## Data Availability

Data is available from the corresponding author on request.

## Footnotes

1 Whilst the UK has been relatively late in adopting emergency alerting, plans to deploy it via CB were initiated in early 2020. The arrival of COVID-19 made this plan both more urgent and more challenging. Deployment of emergency alerting is, at the time of publication, still awaited (Hansard online 2021).

2 The authors acknowledge that there is considerable debate about the best way of describing ethnic diversity, and adopted this terminology as it is that currently used by the UK Environment Agency (DEFRA, 2020).
